# Adherence to Antidiabetic Medications among Women with Gestational Diabetes

**DOI:** 10.1155/2021/9941538

**Published:** 2021-08-06

**Authors:** Michelle Asiedu-Danso, Irene A. Kretchy, Jeremiah Kobby Sekyi, Augustina Koduah

**Affiliations:** Department of Pharmacy Practice and Clinical Pharmacy, School of Pharmacy, College of Health Sciences, University of Ghana, P. O. Box LG, 43 Legon, Ghana

## Abstract

**Background:**

Optimal adherence to prescribed medications in women with gestational diabetes is relevant for perinatal outcomes.

**Objective:**

To summarize available information on the prevalence and factors contributing to medication adherence in women with gestational diabetes from the biological and psychosocial perspectives.

**Methods:**

A literature search on adherence in gestational diabetes was conducted in PubMed/MEDLINE, CINAHL, Scopus, and the Directory of Open Access Journals for studies published on the topic. The Arksey and O'Malley framework for scoping reviews was used to explore and summarize the evidence.

**Results:**

A total of 2395 studies were retrieved of which 13 fully met the eligibility criteria. The studies were reported in Zimbabwe (*n* = 5), Iran (*n* = 1), Mexico (*n* = 1), South India (*n* = 1), the United States of America (*n* = 4), and one multinational study covering Australia, Europe, North and South America. The main types of antidiabetic medications used were insulin (*n* = 6), metformin (*n* = 4), and glyburide (*n* = 2). The prevalence of adherence ranged from 35.6% to 97%, with the assessment tool being self-report measures (*n* = 8). The main factors associated with nonadherence included worsening pregnancy symptoms, side effects of medications, perceived risks, mental health symptoms, poor social support, and socioeconomic status. Recommendations that evolved from the studies to improve adherence included education, counselling, improved support networks, and social interventions, while the main reported interventional study employed continuous education on the impact of adherence on perinatal outcomes.

**Conclusion:**

Medication nonadherence in gestational diabetes seems to be influenced by multiple factors with some educational interventions positively impacting adherence behaviours. Thus, future research in women with gestational diabetes could consider interventions from a multifactorial perspective to improve therapeutic outcomes.

## 1. Introduction

Gestational diabetes is defined as the onset of glucose intolerance during the period of pregnancy [1]. It is associated with diabetes initially recognized in pregnancy and usually resolves when the pregnancy ends [2]. Gestational diabetes is a major public health problem affecting approximately 15.1% of people globally with severe implications on both maternal and neonatal outcomes when left untreated [3–5]. Research on gestational diabetes suggests a longer-term risk of developing Type II diabetes in mothers compared with those without pregnancy-related blood glucose problems [3]. Aside from the conventional effects of diabetes, there have been reports of long-term postpartum diabetes in mothers mainly due to diet and obesity [5]. The risk for gestational diabetes has been linked to women with psychotic disorders during pregnancy and those using specific antipsychotic agents as well [6, 7]. Despite the effects on the mother, gestational diabetes is also associated with adverse outcomes for the baby including neonatal hypoglycemia, jaundice, and respiratory distress syndrome with long-term effects on their health [5].

Gestational diabetes is managed with conventional medications like insulin and oral antidiabetics such as metformin, in addition to diet and exercise [5, 8]. Due to the risks of adverse consequences in pregnancy, management of gestational diabetes requires adequate adherence to these medications and regular clinical appointments [9]. However, poor adherence has been reported and reasons such as mistrust in the safety of medications during pregnancy and fear of birth defects have been implicated [10]. Poor adherence to medications is common and is associated with high morbidity and mortality rates, as well as threats to high economic and logistical burden on public health systems through poor maternal and neonatal outcomes [11, 12].

Previous research or reviews have however focused on the role of diet and weight management in gestational diabetes [13] or on treatment strategies and guidelines [14], while a recent review, for example, has documented only studies relating to a plant-based diet and their impact on gestational diabetes [8].

This scoping review therefore aimed to provide an overview of medication adherence in gestational diabetes to inform future research and provide direction to healthcare professionals, patients, and policymakers on how to increase adherence and health outcomes.

## 2. Methods

A scoping review was conducted following the framework by Arksey and O'Malley, to explore and summarize evidence on medication adherence in gestational diabetes [15]. The process followed the six-stage methodological framework on the identification of research question, identification of relevant studies, selection of studies, data charting, data synthesis, collating, summarising, and reporting. The sixth stage which involved stakeholder consultations was however not utilized in this review.

The review protocol was registered in Open Science Framework (https://osf.io/vfp7n), and the Preferred Reporting Items for Systematic Reviews and Meta-Analysis extension for Scoping Reviews (PRISMA-ScR) was adopted in the reporting [16].

### 2.1. Step 1: Research Questions

The scoping review focused on identifying the area of adherence-related issues in the management of gestational diabetes and was guided by the research question, “What is known about medication adherence and associated factors in women with gestational diabetes?”. Four specific areas of relevance are based on the concept of adherence [17]. (i) What is the rate of medication nonadherence in women with gestational diabetes? (ii) What are the assessment tools used to estimate medication adherence in women with gestational diabetes? (iii) What are the factors associated with medication nonadherence in women with gestational diabetes from the biological and psychosocial perspectives? (iv) What interventions have been utilized in improving medication adherence in women with gestational diabetes?

### 2.2. Step 2: Search Strategy

A comprehensive literature search in MEDLINE (PubMed), CINAHL, and Scopus was performed for all studies published on medication adherence in gestational diabetes. The Directory of Open Access Journals was also searched for grey literature which may not be indexed in the databases listed. The search terms related to adherence to antidiabetic medications and gestational diabetes using keywords, synonyms, and MeSH terms: Adherence, Non-adherence, Non adherence, Compliance, Non-compliance, Non compliance, AND Gestational Diabetes, Diabetes, Pregnancy Induced, Gestational Diabetes Mellitus, Pregnancy-Induced Diabetes, Diabetes Mellitus, Gestational, Diabetes, Pregnancy-Induced, AND Medications, Drugs, Antidiabetic.

### 2.3. Step 3: Screening and Study Selection

Studies were included if they described the prevalence, factors, and/or interventions for medication adherence in gestational diabetes and recommendations. Studies that did not meet these eligibility criteria, as well as reviews, commentaries, and guidelines, were excluded. The eligibility criteria included the mention of adherence to antidiabetic medication in the study. Titles and abstracts of the publications were independently screened by two members of the review team (M.A.D. & I.A.K.). Full-text articles after the initial screening were read. To reduce the potential for selection bias, the screening process was undertaken in duplicate by two reviewers working independently. Disagreements on the eligibility of articles were resolved through discussions.

### 2.4. Step 4: Data Charting

The data were organized based on information on the authors of the publication, year of publication, the title of publication, country of study, study type, objectives, adherence measures, other outcome variables, and key findings.

### 2.5. Step 5: Collating, Summarizing, and Reporting Results

After charting the data, results were summarized in line with the research questions where information on prevalence, adherence assessment, associated factors, and interventions relating to medication adherence in women with gestational diabetes and recommendations were noted. In reporting the results, the pharmaceutical care, clinical, and policy implications were also suggested.

## 3. Results

**Figure 1:**
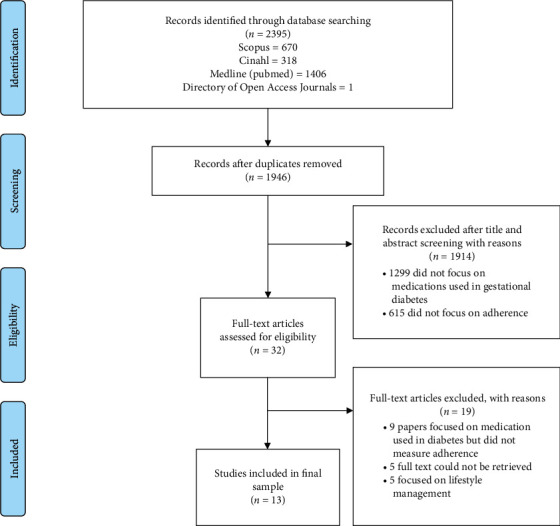
Study selection flow chart.

The initial search of electronic databases yielded 2395 citations of which 1946 remained after removing 449 duplicates. After reading through the titles and abstracts of the 1946 citations, 32 were selected for full-text review and assessment for eligibility. A total of 13 journal articles were deemed eligible and were included in this review. Most of the studies that were excluded at the full-text review were studies that focused on the medication used in diabetes but did not measure adherence (*n* = 9), focused on adherence to lifestyle therapy (*n* = 5), or could not be retrieved (*n* = 5).  Figure 1 presents the PRISMA-ScR diagram indicating the selection of the publications.

The papers covered Europe, Asia, Africa, North and South America, and Australia (Table 1). 12 papers were published in the last 7 years (between 2014 and 2020), and only one was published in 1990. All the studies were situated in specialist settings such as antenatal clinics or diabetic clinics. Two studies were interventional, focusing on analysing the association between adherence to antidiabetic therapy (diet, physical activity, and medications) and perinatal outcomes [18] and the association between antidiabetic therapy and glycaemic control [19].

### 3.1. Types of Medications and Adherence Rates

The dominant medications used in the management of gestational diabetes according to the papers reviewed were insulin [18, 20–24] and metformin [19, 21, 22, 25]. Besides medications, adherence to lifestyle/behaviours like self-monitoring of blood glucose levels, dietary therapy, and physical activity were considered [18, 20, 22–28]. Self-report measures were the recorded means of estimating adherence [18–26, 28, 29]. Medication adherence ranged between 35.6% and 97% (Table 2).

### 3.2. Factors Associated with Medication Adherence in Gestational Diabetes

From this scoping review, the factors associated with medication adherence in gestational diabetes were categorised under biological and psychosocial factors.

#### 3.2.1. Biological Factors

Biological factors that impacted medication adherence included pathophysiology of diabetes [18], effects of pregnancy such as vomiting, loss of appetite, unusual discomfort [18, 26, 30], complicated medication regimen [26, 30], the type of medications used [24], and medication side effects [25].

#### 3.2.2. Psychosocial Factors

Psychosocial factors reported to negatively impact medication adherence included patients' beliefs [20], fear of disease and medication complications [21], beliefs in abstaining from medication while pregnant despite being ill and belief in the use of herbal remedies when pregnant [29], concerns for the fetus health and wellbeing [21, 23, 29], poor socioeconomic status, lack of support from significant others and peers [22–24, 26, 28], poor health information [27, 30], and financial barriers [26] (Table 3).

## 4. Discussion

The review identified 13 papers that reported on medication adherence in gestational diabetes. The prevalence of adherence ranged from 35.6% to 97% with worsening pregnancy symptoms, side effects of medications, perceived risks, poor social support, and socioeconomic status as the reported factors associated with nonadherence.

Although self-reported measures were the main tools for estimating medication adherence in this review [18–26, 28, 29], some studies assessed fasting blood glucose or glycated haemoglobin as a means of confirming adherence and predicting disease outcomes [18, 22, 28]. Generally, adherence to oral antidiabetic medications and insulin has been found to range between 36-93% and 62-64%, respectively [31, 32]. This review observed consistent levels of adherence to oral hypoglycaemic medication and insulin which averaged 86% and 64%, respectively [18–22, 24, 25]. These reported levels could be associated with the population used in the study where women have been shown to be less adherent to medication than men in diabetes management. Furthermore, pregnant women are less adherent to medication due to worsening pregnancy symptoms, side effects of medications, perceived risks to the unborn child, and mental health issues such as anxiety and depression [18, 24–26, 30]. These documented factors are consistent with observations from the review. Other biological barriers to adherence from the review include complications and complex medication regimen [26, 30]. These factors may have reduced patient tolerance to medication and led to women forgetting to take their medications or decrease their motivation to take their medications, further reducing adherence [33, 34].

The main psychosocial factor influencing adherence in the papers reviewed was social support [22–24, 26, 28]. The role of support from family and significant others in providing monitoring, reassurances, and coping avenues for patients to deal with their health-related concerns and its impact on adherence cannot be overlooked in diabetes management [35]. Some papers reviewed described supportive behaviours such as peer groups for pregnant women with diabetes, spousal accompaniment to antenatal clinics, and understanding from family members [22–24, 26, 28]. Supportive behaviours such as these have been reported to positively impact medication adherence in diabetes management [35, 36]. Financial support especially from friends also plays a huge role in improving adherence [37]. Papers reviewed showed a similar trend. Pregnant women with poor financial support were less adherent compared to women with better financial support. This is because women with poor financial support could not attend antenatal clinic regularly or purchase medications to ensure their availability and adherence [30, 38]. Financial support from family and friends is vital especially in low-income settings where poor adherence rates have been reported because patients could not afford their medications [38–40]. The impact of social support and the potential to the use of a support network in improving diabetes medication adherence among pregnant women especially in low- and middle-income settings is highly recommended based on findings from this review.

Some papers also showed that patients with high socioeconomic status (SES) had higher adherence rates [20, 25, 27], while another study showed the opposite [20]. Women with low socioeconomic status were often nonadherent due to financial constraints. Thus, when they received medication subsidies and improved access to healthcare for instance, through insurance schemes, they were more likely to be adherent [25, 30, 41]. This correlates with literature which demonstrates an increased adherence behaviour with health insurance [40, 42]. Meanwhile, according to Haghdoost et al. [20], women with higher SES were nonadherent due to lifestyle concerns such as having a demanding job which negatively impacted on their adherence behaviour. These women were often burdened with work responsibilities and fixed schedules that either made them forget to take their medications or decide to skip them. On the other hand, those with low SES often had more flexible and less demanding jobs and could make time to take their medications. Again, the women with high SES had better health literacy and were not concerned with their diagnosis while those with low SES were very disturbed about their diagnosis and the likely financial costs of disease complications due to nonadherence [20]. These findings are however contrary to available literature on medication adherence and socioeconomic status among persons with diabetes mellitus generally [43, 44]. The association between socioeconomic status and medication adherence among women with gestational diabetes needs to be further studied to identify peculiarities that might be useful for improving medication adherence.

Poor information retention was cited as a cause of medication nonadherence by Mukona et al. [18]. Poor information retention was reportedly caused by health professionals who overload patients with too much health information on a visit. Poor communication from the health professional included failure to communicate clearly, inept health advice due to lack of expertise with the disease, and long waiting times due to inadequately qualified staff [27, 30]. This finding is consistent with literature [45, 46]. Thus, the need for continuous education from qualified health professionals cannot be overemphasized based on these findings.

In terms of interventions for adherence, some studies demonstrated the positive associations between medication knowledge and adherence and improved disease outcome [19, 21, 22, 27, 30]. Some studies instituted continuous adherence training through health professionals, while others were through peer support groups or community champions [18, 19, 22, 27, 30]. Providing disease and medication knowledge improved health literacy, dispelled myths and negative beliefs, set medication side effect expectations, and allayed fears and concerns with medication use. These findings have been corroborated in literature [47–49]. These studies demonstrated the importance of continuous education of patients especially in the management of chronic diseases.

Identifying the factors for adherence behaviour can be leveraged to designing policies and frameworks to manage gestational diabetes and increase adherence among pregnant women. Medication adherence is complex, requiring multifactorial strategies to improve and promote it. Thus, the application of nonresource intensive interventions from multiple perspectives could be used to enhance medication adherence. This will be useful in the proper and effective management of gestational diabetes and improve the perinatal and neonatal outcomes of patients with gestational diabetes. In terms of clinical practice and policy, patient counselling and education could be targeted towards patients based on their health and social groupings. This will be key to tailoring patient counselling to meet the patient type and hence enhance adherence. Also, policies designed could consider addressing patient barriers to adherence.

Despite the above, this review acknowledges the limitation that some studies and grey or unpublished literature may have been missed, because they may not be indexed in the databases that were utilised. Second, since the aim of this review was to scope available evidence on medication adherence in gestational diabetes, quality appraisal of the included studies was not conducted. In addition, most reported studies in literature focused on adherence to nutritional and physical exercise regimens for pregnant women, and few studies have specifically reported on medication adherence in women with gestational diabetes. Thus, this scoping review observes the gap in gestational diabetes medication adherence research and the opportunity to address barriers to improve medication adherence.

## 5. Conclusion

Medication adherence in gestational diabetes seems to be influenced by factors from a biopsychosocial perspective with some educational interventions positively impacting adherence behaviours. The review observed complex factors that influence patients' medication adherence in gestational diabetes. Thus, future research in women with gestational diabetes could consider interventions from a multifactorial perspective to improve therapeutic outcomes.

## Figures and Tables

**Table 1:** Summary of studies on medication adherence in patients with gestational diabetes.

Study	Title	Country	Study type	Outcome
Mokena et al. (2018) [18]	Association between adherence to anti-diabetic therapy and adverse maternal and perinatal outcomes in diabetes in pregnancy	Zimbabwe	Cohort study (intervention)	Perinatal outcomes
Mokena et al. (2017) [26]	Barriers of adherence and possible solutions to non-adherence to antidiabetic therapy in women with diabetes in pregnancy: patients' perspective	Zimbabwe	Descriptive qualitative study	N/A
Haghdoost et al. (2019) [20]	The impact of socioeconomic factors on the adherence of patients with gestational diabetes mellitus to medical recommendations	Iran	Prospective study	N/A
Chávez García et al. (2019) [21]	Gestational diabetes adherence to treatment and metabolic control	Mexico	Cross-sectional Study	Glycemic control
Lupattelli et al. (2014) [29]	Adherence to medication for chronic disorders during pregnancy: results from a multinational study	Europe, North and South America, and Australia	Multinational, cross-sectional study	N/A
Krishnakumar et al. (2020) [19]	Impact of patient education on KAP, medication adherence and therapeutic outcomes of metformin versus insulin therapy in patients with gestational diabetes: a hospital based pilot study in South India	South India	Prospective observational (intervention)	Glycemic control and knowledge, attitude, and practice of medication adherence
Mukona et al. (2017) [27]	Barriers and facilitators of adherence to antidiabetic therapy in pregnant women with diabetes: Health care workers' perspectives	Zimbabwe	Descriptive study	N/A
Mukona et al. (2017) [30]	Development of an adherence promotion framework for women with diabetes in pregnancy to improve adherence to anti-diabetic therapy and perinatal outcomes	Zimbabwe	Mixed methods sequential dominant status	Perinatal outcomes
Mukona et al. (2017) [28]	Adherence to anti-diabetic therapy in women with diabetes in pregnancy	Zimbabwe	Descriptive study	Perinatal outcomes
Refuerzo et al. (2015) [25]	The effects of metformin on weight loss in women with gestational diabetes: a pilot randomized, placebo-controlled trial	United States of America	Randomized controlled trial	Gestational weight gain
Ruggiero et al. (1990) [23]	Impact of social support and stress on compliance in women with gestational diabetes. Diabetes care	United States of America	Cross-sectional	Adherence
Sperling et al. (2018) [24]	Prenatal care adherence and neonatal intensive care unit admission or stillbirth among women with gestational and preexisting diabetes mellitus	United States of America	Retrospective cohort	Perinatal outcomes
Carter et al. (2020) [22]	Pilot randomized controlled trial of diabetes group prenatal care	United States of America	Randomized controlled trial	Perinatal outcomes

N/A: not available.

**Table 2:** Adherence levels per study and recommendations/outcomes recorded.

Study	Type of measure	Level of adherence	Interventions made	Study recommendations to improving adherence
Mokena et al. (2018) [18]	Self-report	68.79%	Continuous education of patients	Advocacy for strict adherence to healthy lifestyle habits to control diabetes mellitus particularly in developing countries like Zimbabwe where access to health care and quality of health care are huge problems.
Mokena et al. (2017) [26]	Self-report	N/A	N/A	Fostering family, peer, and community support, getting financial support, and improvement of service at the hospital
Haghdoost et al. (2019) [20]	Self-report	48.90%	N/A	Educating target groups and doing social interventions.
Chávez García et al. (2019) [21]	Self-report	90% for metformin cohort and 71% for insulin cohort	N/A	Training patients with diagnosis of gestational diabetes and emphasize the appropriate adherence to the treatment established.
Lupattelli et al. (2014) [29]	Self-report	37%	N/A	Adequate counselling and proper teratogenic risk communication to potentially attenuate women's negative beliefs about medication and heighten medication adherence during pregnancy.
Krishnakumar et al. (2020) [19]	Self-report	5.6+/-1.15	Continuous patient education	Continuous patient education to positively impact on the knowledge, attitude, practice, and medication adherence patterns of pregnant women with gestational diabetes.
Mukona et al. (2017) [27]	N/A	N/A	N/A	Subsidizing healthcare costs, collaboration among health care workers, and establishment of a unit dedicated to care of pregnant women with diabetes
Mukona et al. (2017) [30]	N/A	35.6%	N/A	Utilization of the framework model designed will improve adherence to antidiabetic therapy and help to reduce incidence of adverse perinatal outcomes.
Mukona et al. (2017) [28]	Self-report	80%	N/A	Customizing health education to suit individual patient needs.
Refuerzo et al. (2015) [25]	Self-report	97%	N/A	Medication side effects and dissatisfaction were the greatest inhibitor of medication adherence.
Ruggiero et al. (1990) [23]	Self-report	71%	N/A	Social support is a particularly important variable to assess when evaluating regimen compliance in pregnant women with gestational diabetes
Sperling et al. (2018) [24]	Self-report	N/A	N/A	Factors that improve prenatal care should be encouraged as it improved perinatal and neonatal outcomes
Carter et al. (2020) [22]	Self-report	6.4+/-1.5	Group care meetings	Most patient's needs can be managed in the group setting with additional individual visits, as needed.

N/A: not available.

**Table 3:** Description of factors associated with medication adherence based on the biopsychosocial perspective.

ID	Biological factors	Psychosocial factors
Mokena et al. (2018) [18]	Unusual pregnancy discomfort	Information overload from health professionals in a short time
Mokena et al. (2017) [26]	Pathophysiology of diabetes, effects of pregnancy, complicated therapeutic regimen	Poor socioeconomic status; lack of family, peer, and community support; cultural and religious beliefs; and poor health care system.
Haghdoost et al. (2019) [20]	N/A	Fear of medication and disease complication, financial barriers, high workload
Chávez García et al. (2019) [21]	N/A	Patient acceptance of route of administration, educational level attained
Lupattelli et al. (2014) [29]	N/A	Personal beliefs (belief in abstaining from medication while pregnant despite being ill, belief in the use of herbal remedies when pregnant)
Krishnakumar et al. (2020) [19]	N/A	Low knowledge levels about the risk factors for gestational diabetes and the course of gestational diabetes. Low knowledge on the increased risk for future type2 diabetes after a previous diagnosis.
Mukona et al. (2017) [30]	N/A	Lack of finances, lack of health education, inadequate expertise of staff
Mukona et al. (2017) [18]	Complications of pregnancy (loss of appetite, nausea), complicated medication regimen	N/A
Mukona et al. (2017) [28]	N/A	Financial challenges, lack of spousal support
Refuerzo et al. (2015) [25]	Medication side effects (diarrhea, nausea, and hypoglycemia), medication intolerance	N/A
Ruggiero et al. (1990) [23]	N/A	Concern for fetus health, social support, stress
Sperling et al. (2018) [24]	Medication used	Previous psychiatric history, previous addictions (tobacco or alcohol use), intimate partner violence, socioeconomic status (health insurance, employment, married or single)
Carter et al. (2020) [22]	N/A	Peer support; reassurance from women on a particular care plan served to encourage those newly rolled on and were apprehensive to adhere to treatment, also, accounts from other women set expectations for medication and lifestyle modification challenges

N/A: not available.
